# Delayed-Onset Immune-Related Colitis Following Pancreaticoduodenectomy in Patients With Gastric Cancer and Pancreatic Invasion Treated With Immune Checkpoint Inhibitors

**DOI:** 10.7759/cureus.68480

**Published:** 2024-09-02

**Authors:** Koshiro Morino, Shu Nagatomo, Kunpei Ishida, Taro Ueo, Takafumi Machimoto

**Affiliations:** 1 Department of Gastroenterological Surgery, Tenri Hospital, Tenri, JPN; 2 Department of Gastroenterology, Tenri Hospital, Tenri, JPN

**Keywords:** immune checkpoint inhibitor, pancreaticoduodenectomy, colitis, delayed onset, immune-related adverse event

## Abstract

Immune checkpoint inhibitors (ICIs) have been approved for treating various advanced malignancies. Immune-related adverse events (irAEs) can manifest diversely and at varying times. However, postoperative diarrhea is a common complication in pancreaticoduodenectomy (PD). This case report presents a unique instance of delayed-onset irAE colitis occurring one year after PD in a patient with gastric cancer who received neoadjuvant nivolumab, a monoclonal antibody targeting human programmed death 1. A 54-year-old male developed severe diarrhea and weight loss, ultimately diagnosed with irAE colitis, which responded to steroid therapy. This report underscores the importance of extended monitoring, recognizing the potential for late-onset toxicities associated with ICIs, and differentiating from PD-related diarrhea.

## Introduction

Immune checkpoint inhibitors (ICIs) have revolutionized cancer therapy, offering new hope for patients with advanced malignancies. By blocking proteins that typically deactivate cytotoxic T cells, these immunotherapeutic agents enhance the immune response against tumor cells [1]. However, ICIs can induce immune-related adverse events (irAEs) affecting various organs, with gastrointestinal toxicity being prevalent [2]. While most irAEs manifest within the first 12 weeks of treatment, diarrhea and colitis often emerge six to seven weeks after ICI administration [3,4]. Notably, delayed-onset irAEs can occur months or even years after therapy, posing significant diagnostic and therapeutic challenges.

Pancreaticoduodenectomy (PD) is the standard surgical treatment for resectable pancreatic head, distal bile duct, or ampullary cancers. It is also an option for gastric cancer with pancreatic invasion [5]. The extent of lymph node and nerve plexus dissection during PD for pancreatic head cancer remains controversial due to the tumor’s aggressive nature [6]. Clearance of the superior mesenteric artery (SMA) plexus can result in refractory postoperative diarrhea [7], impacting quality of life and delaying or interrupting adjuvant chemotherapy.

This case report presents a unique instance of delayed-onset irAE colitis occurring one-year post-PD in a patient with gastric cancer treated with neoadjuvant nivolumab. This case underscores the importance of prolonged surveillance for potential late-onset toxicities associated with ICIs.

## Case presentation

A 54-year-old man presented to our hospital with anorexia, abdominal distension, and a 20-kg weight loss. The patient presented with mild anemia, and carbohydrate antigen 19-9 (CA19-9) was elevated at 473.1 U/mL. Laboratory investigations are shown in Table 1.

**Table 1:** Laboratory results before treatment and one year after the final administration Result 1: before treatment, Result 2: one year after the final administration

Test	Result 1	Result 2	Normal range
Hemoglobin	11.1 g/dL	11.6 g/dL	13.7-16.8 g/dL
Mean corpuscular volume	91.1 fL	88.0 fL	83.6-98.2 fL
Platelets	16.5 × 10^4^/µL	25.9 × 10^4^/µL	15.8-34.8 × 10^4^/µL
White blood cell	4.04 × 10^3^/µL	7.09 × 10^3^/µL	3.3-8.6 × 10^3^/µL
Total bilirubin	0.6 mg/dL	0.5 mg/dL	0.4-1.5 mg/dL
Alanine transaminase	21 U/L	22 U/L	10-42 U/L
Alkaline phosphatase	47 U/L	84 U/L	38-113 U/L
Aspartate transaminase	24 U/L	21 U/L	13-30 U/L
Lactate dehydrogenase	186 U/L	123 U/L	124-222 U/L
Blood urea nitrogen	20.1mg/dL	15.1 mg/dL	8-20 mg/dL
Creatinine	0.8 mg/dL	0.8 mg/dL	0.7-1.1 mg/dL
Total protein	6.9 g/dL	7.2 g/dL	6.6-8.1 g/dL
Albumin	4.2 g/dL	3.7 g/dL	4.1-5.1 g/dL
Activated partial thromboplastin clotting time	29.6 seconds	28.7 seconds	23-35 seconds
Prothrombin time	12.4 seconds	12.4 seconds	10.5-13.3 seconds
International normalized ratio	1.3	1.07	0.8-1.2
Free thyroxine	1.26 ng/dL	1.20 ng/dL	0.93-1.70 ng/dL
Thyroid-stimulating hormone	2.630 mIU/L	0.693 mIU/L	0.61-4.23 mIU/L
Carcinoembryonic antigen	2.3 ng/mL	0.6 ng/mL	<5 ng/mL
Carbohydrate antigen 19-9	473.1 U/mL	9.0 U/mL	<37 U/mL

**Figure 1:**
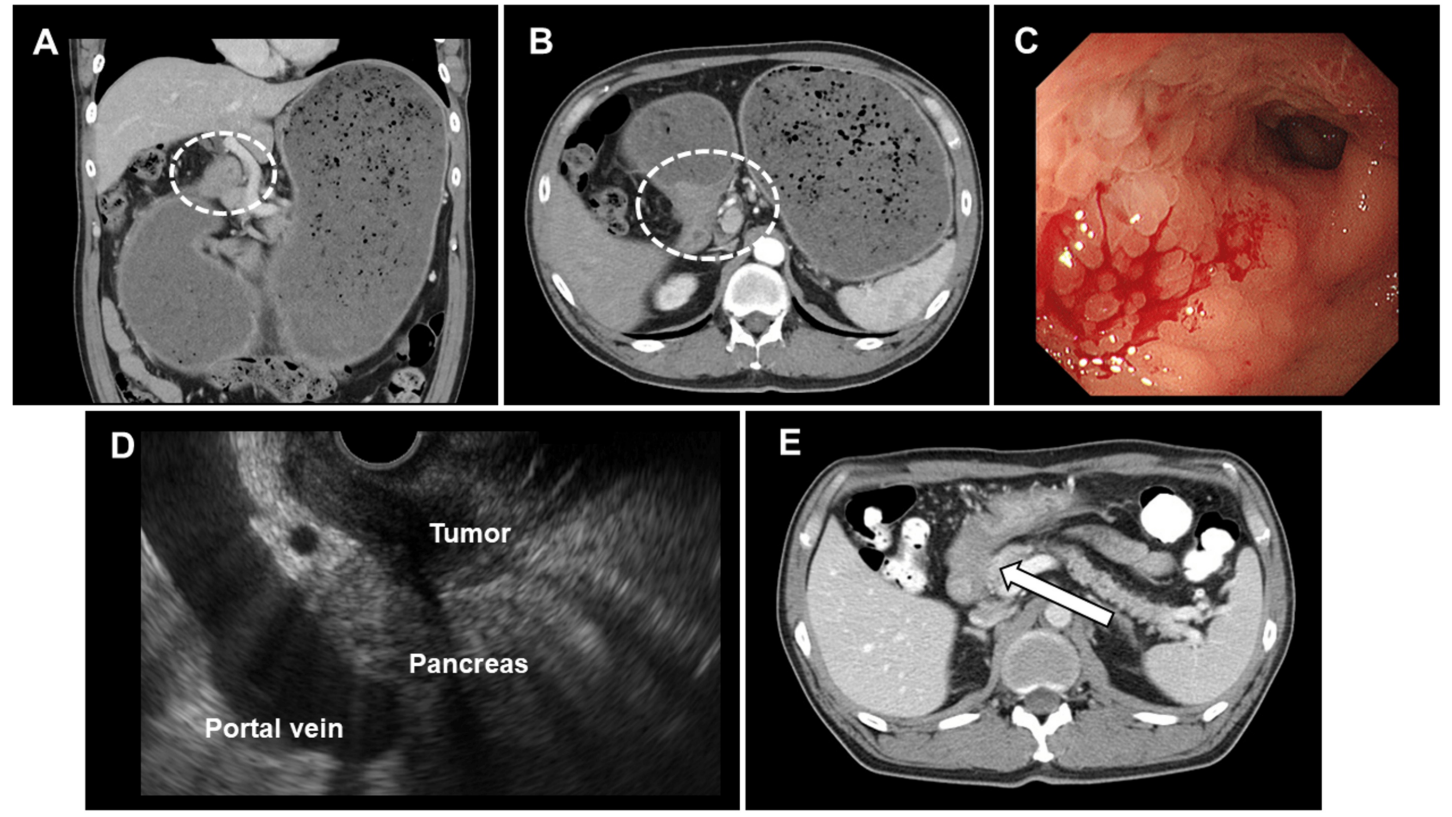
Pre-treatment imaging and endoscopic findings Initial contrast-enhanced abdominal computed tomography (CT) in the coronal (A) and axial (B) planes demonstrates significant gastric residue and pyloric wall thickening (dashed lines). Initial endoscopy (C) reveals a circumferential type 3 tumor near the pylorus, causing pyloric stenosis, marked gastric retention, and duodenal extension. Endoscopic ultrasound (D) shows a large, hypoechoic area in the stomach and duodenum with indistinct pancreatic head borders. Post-decompression CT (E) depicts extensive tumor involvement from the pylorus to the duodenal bulb, with suspected pancreatic head invasion (white arrow).

A computed tomography (CT) scan revealed an extensive tumor extending from the gastric pylorus through the descending duodenum to the pancreatic head, causing pyloric stenosis and marked gastric distension (Figures 1A, 1B). Upper gastrointestinal endoscopy demonstrated a significant amount of food residue within the stomach, edematous gastric mucosa, and a tumor-induced narrowing of the pyloric ring that precluded scope passage (Figure 1C). Following endoscopic removal of the residue and gastric lavage, repeat endoscopy and pathological examination were performed. While the mucosal surface was devoid of malignant cells, endoscopic ultrasound-guided fine needle aspiration identified adenocarcinoma, establishing a diagnosis of "gastro-duodenal" cancer with pancreatic head invasion (Figure 1D). Differentiating between gastric cancer and duodenal cancer was not possible based on imaging and histopathological evaluation. A post-decompression CT scan confirmed extensive tumor involvement from the pylorus to the duodenal bulb, with suspected pancreatic head infiltration (Figure 1E). Further evaluations revealed no unresectable factors such as distant metastasis, rendering PD a feasible option. However, given the advanced local disease and poor nutritional status, gastric bypass followed by neoadjuvant chemotherapy was elected.

**Figure 2:**
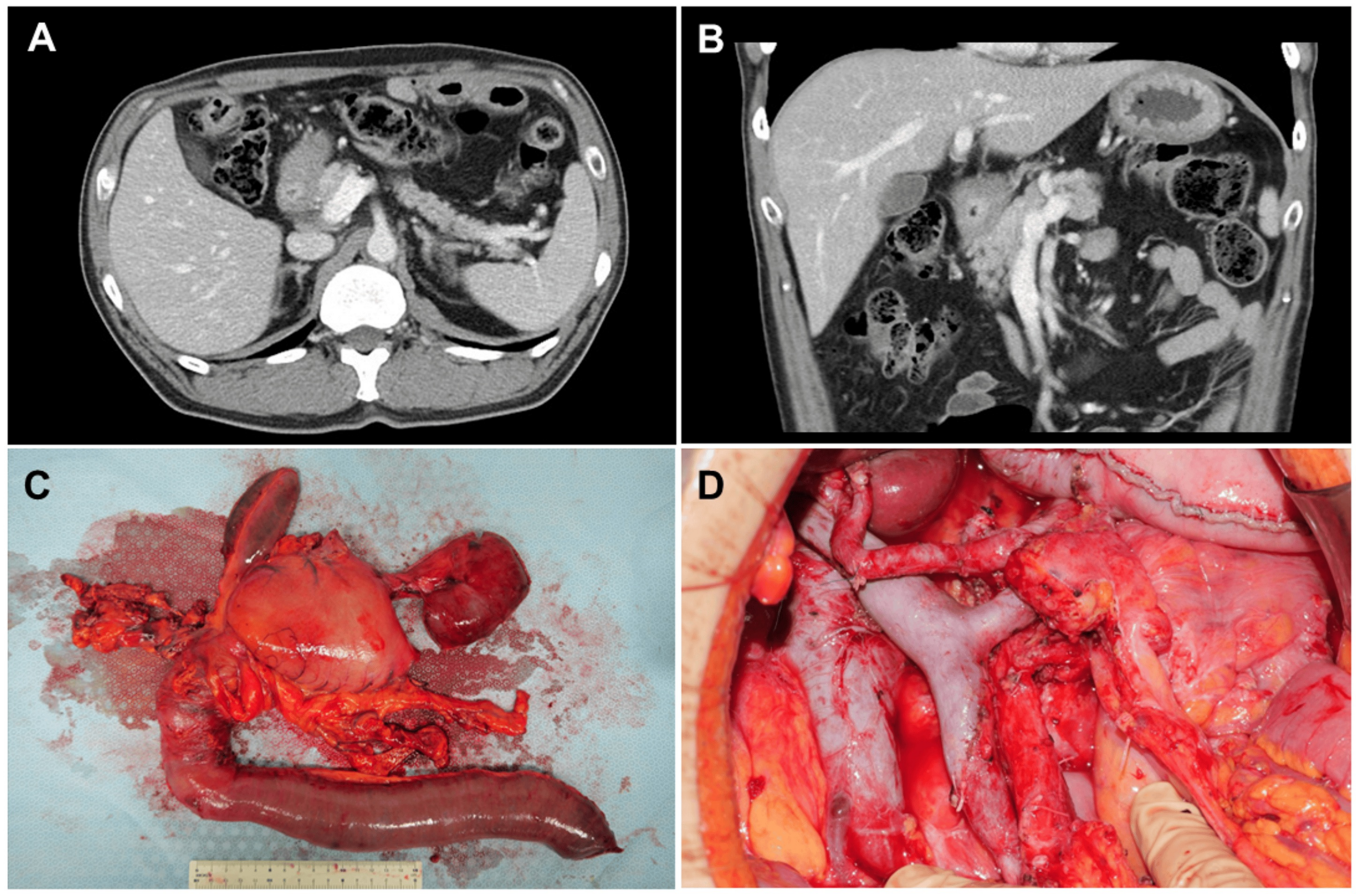
Post-chemotherapy imaging and surgical findings Post-chemotherapy contrast-enhanced CT in the axial (A) and coronal (B) planes shows a tumor with slightly decreased enhancement but unchanged size. A photograph of the resected specimen (C) demonstrates the tumor near the pylorus with pancreatic invasion. Post-pancreatoduodenectomy with D2 lymph node dissection (D) reveals preservation of the superior mesenteric artery nerve plexus.

A laparoscopic gastrojejunostomy connecting the greater curvature of the stomach to a point 20 cm distal to the ligament of Treitz was performed without complications, enabling oral intake. Two weeks later, systemic therapy with S-1 (80 mg/m²/day on days 1-14), oxaliplatin (130 mg/m²), and nivolumab (360 mg/body) was initiated every three weeks for four cycles, without significant adverse events. Four weeks post-treatment, preoperative CT demonstrated no tumor growth but reduced contrast enhancement (Figures 2A, 2B), while the surrogate tumor marker CA19-9 declined from 473.1 to 42.0 U/mL. The surgical procedure involved a PD with D2 lymph node dissection, following protocols for both duodenal and gastric cancers (Figure 2C). Due to the tumor's aggressive infiltration, portions of the first and second segments of the pancreatic nerve plexus, and the right side of the celiac nerve plexus were resected, preserving the SMA nerve plexus circumferentially (Figure 2D).

Reconstruction encompassed gastropancreatic anastomosis, choledochojejunostomy, and gastrojejunostomy. Postoperatively, the patient experienced diarrhea seven times daily, which resolved with codeine phosphate and loperamide. No other complications arose, and the patient was discharged on postoperative day 20.

Pathological examination revealed a tumor spanning the gastric and duodenal junction, making a precise distinction challenging, diagnosed as gastric cancer with duodenal and pancreatic head invasion. The final diagnosis was L, type 3, 45 × 30 mm, tub1>tub2, pT4b (pancreas), Ly0, V0, pN1 (1/50), pPM0, pDM0, with a clinical post-chemotherapy histological therapeutic effect of grade 1b, resulting in T4bN1M0 stage IIIB in accordance with the 15th Edition of the Japanese Classification of Gastric Cancer. Due to patient refusal, postoperative adjuvant chemotherapy was omitted.

**Figure 3:**
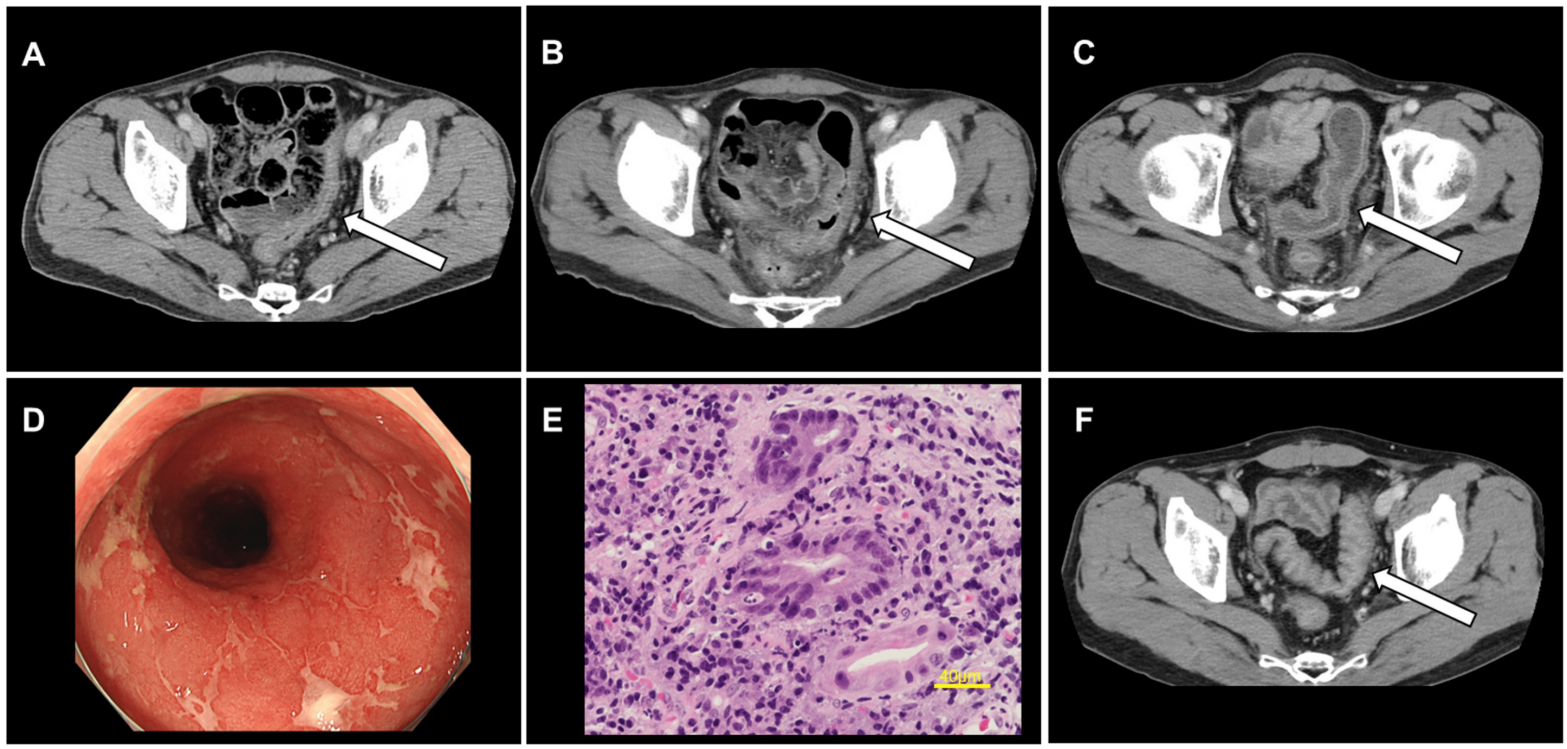
Longitudinal imaging and pathological findings of colitis following surgery Postoperative follow-up contrast-enhanced CT at three months (A), six months (B), and one year (C) demonstrated progressive thickening of the sigmoid colon (white arrow). Rectal images from colonoscopy (D) show a rough mucosa with decreased vascular pattern throughout the colon, consistent with colitis. The sigmoid colon and rectum exhibit scattered erosions and severe inflammation. Pathological biopsy (E) reveals reduced glandular structures, abundant granulation tissue, inflammatory cell infiltration, and cryptitis. At two years postoperatively, contrast-enhanced CT (F) shows improvement in sigmoid colon wall thickening (white arrow).

Approximately two months post-discharge and three months after the final nivolumab dose, the patient experienced worsening diarrhea, which improved with opioid antidiarrheal medications. Follow-up CT scans at three and six months post-surgery revealed no evidence of recurrence or abnormalities in the colon (Figures 3A, 3B). However, 13 months post-surgery (14 months after the final nivolumab dose), the patient developed severe diarrhea, occurring up to 20 times daily, accompanied by a 15-kg weight loss since surgery. A CT scan demonstrated localized colonic wall thickening in the descending and sigmoid colon (Figure 3C), while lower gastrointestinal endoscopy revealed scattered colonic ulcers and erosions (Figure 3D). Biopsies confirmed ulcerative granulation tissue with cryptitis (Figure 3E), consistent with a diagnosis of grade 3 immune-related colitis [8].

Treatment was initiated with intravenous prednisolone at 30 mg/day, which was escalated to 50 mg/day after one week due to persistent symptoms. Clinical improvement followed, allowing for prednisolone tapering by 10 mg weekly and transition to oral administration at 30 mg. At discharge, the patient had gained 10 kg. Steroids and antidiarrheal medications were discontinued four months post-treatment, with an additional 5-kg weight gain. A contrast-enhanced CT scan performed two years postoperatively demonstrated improvement in sigmoid colonic wall thickening (Figure 3F). The patient remained asymptomatic and recurrence-free at three-year and two-and-a-half-year follow-ups, respectively.

## Discussion

This is the first reported case of late-onset irAE colitis following PD in a patient treated with ICIs as neoadjuvant chemotherapy. While irAEs typically manifest within three months of therapy initiation [9], delayed-onset irAEs have been reported more than three months to a year after treatment cessation; however, the definition remains unclear. Table 2 summarizes irAE colitis data from ICI clinical trials [3,4,10-15], indicating a colitis incidence of approximately 10-20%, with grade 3 or higher colitis occurring in 1-4% of cases. The median onset is typically two and five months; however, delayed-onset irAE colitis, as observed in this case, occurs in around 3% of patients, with grade 3 colitis affecting 1-2%. Given the limited long-term adverse event monitoring in these trials, the true incidence of delayed-onset irAEs may be underestimated.

**Table 2:** Immune-related adverse event severe diarrhea/colitis according to category and grade of previous trials ICI, immune checkpoint inhibitor; IrAE, immune-related adverse event; NSCLC, non-small-cell lung cancer; PD, pancreaticoduodenectomy

Trial, author	Agent	Indication	All grades (%)	≥Grade 3 (%)	Median time to onset	Delayed onset (months)	Delayed onset ≥grade 3 (%)
CheckMate-238, Mandala et al. [2]	Anti-PD-1	melanoma	24.8%	0.4%	8 weeks	7.3% (3-12 months)	0% (3-12 months)
ONO-4538-02, ONO-4538-08, Uhara et al. [10]	Anti-PD-1	melanoma	10.2%	3.4%	-	-	-
ONO-4538-05, ONO-4538-06, Horinouchi et al. [11]	Anti-PD-1	NSCLC	8.1%	0%	5.59 months	-	-
ONO-4538-15 CheckMate 205, Ghisoni et al. [12]	Anti-PD-1	Hodgkin lymphoma	11.8%	0%	-	-	-
ONO-4538-12 ATTRACTION-2, Nuzzo et al. [13]	Anti-PD-1	Gastric cancer	7%	1%	-	-	-
Meta-analysis, Ghisoni et al. [12]	All ICIs	Melanoma, lung cancer	17.9%	-	42 days	-	-
Multicenter cohort study, Owen [3]	Anti-PD-1 ± anti-CTLA4	Melanoma	-	-	5 months	31/999 (>12 months)	18/999 (>12 months)
Cohort study, Nuzzo et al. [13]	Anti-PD-1/L1	Renal and urothelial cancer	7.4%	3.8%	17 weeks	5/470 (>12 months)	-
Review, de La Rochefoucauld et al. [4]	Anti-PD-1	All	10-15%	<2%	-	-	-

Colitis is a common irAE, and refractory diarrhea warrants consideration long after ICI discontinuation. PD can also induce refractory diarrhea, with its incidence significantly influenced by the extent of SMA plexus dissection [6,7]. While approximately 10-12% of patients with preserved SMA experience postoperative diarrhea [6,7], significantly higher than the incidence of delayed-onset irAE colitis, the timing of onset is crucial for differential diagnosis. Unlike typical postoperative diarrhea, which often resolves within the initial postoperative period, this case demonstrated persistent and worsening diarrhea requiring long-term opioid antidiarrheal treatment. Therefore, as PD-related diarrhea does not involve colitis, CT imaging and histopathological evaluation via endoscopic biopsy are useful for differential diagnosis.

PD remains the gold standard for resectable pancreatic head, distal bile duct, and ampullary cancers. It is also indicated for advanced gastric cancer with pancreatic invasion, absent distant metastasis [5], as in this case. The increasing use of ICIs in treating various malignancies has led to their incorporation into standard first-line therapy for unresectable gastric [16] and bile duct cancers [17], with ongoing clinical trials for pancreatic cancer. Neoadjuvant therapy, supported by robust evidence for advanced gastric cancer, is the standard treatment for pancreatic cancer [18] and gaining prominence in bile duct cancer. Consequently, the incidence of PD following neoadjuvant ICI therapy is anticipated to rise.

This case highlights the unexpected absence of recurrence despite advanced disease, as long-term outcomes after PD for such cases, particularly with lymph node metastasis, remain unfavorable [19]. Despite being at a high risk, our patient achieved a three-year recurrence-free survival. The association between delayed-onset irAEs and prolonged survival [20] suggests extended susceptibility to irAEs, necessitating vigilant long-term monitoring.

## Conclusions

This report presents a rare case of late-onset irAE colitis one-year post-PD in a patient with advanced gastric cancer with pancreatic invasion treated with neoadjuvant nivolumab. Clinicians should maintain a high index of suspicion for diarrheal symptoms as a potential late manifestation of ICI therapy, even long after treatment completion.
